# 3D Geometric Morphometric Analysis of Calcaneal Morphology in Domestic Caprinae: Sheep (*Ovis aries*) and Goat (*Capra hircus*)

**DOI:** 10.3390/ani15040556

**Published:** 2025-02-14

**Authors:** Barış Can Güzel, Tomasz Szara, Burak Ünal, Sokol Duro, Fatma İşbilir, Funda Yiğit, Mihaela-Claudia Spataru, Karolina Goździewska-Harłajczuk, Ozan Gündemir

**Affiliations:** 1 Department of Anatomy, Faculty of Veterinary Medicine, Siirt University, 56100 Siirt, Türkiye; bcguzel@hotmail.com (B.C.G.); fatmaisbilir42@gmail.com (F.İ.); 2 Department of Morphological Sciences, Institute of Veterinary Medicine, Warsaw University of Life Sciences-SGGW, 02-776 Warsaw, Poland; 3 Department of Anatomy, Faculty of Veterinary Medicine, Istanbul University-Cerrahpasa, 34320 Istanbul, Türkiye; burak.unal@iuc.edu.tr; 4 Department of Anatomy, Faculty of Veterinary Medicine, Agricultural University of Tirana, 1000 Tirana, Albania; durosokol@ubt.edu.al; 5 Department of Histology and Embryology, Faculty of Veterinary Medicine, Istanbul University-Cerrahpasa, 34320 Istanbul, Türkiye; fyigit@iuc.edu.tr; 6 Department of Public Health, Faculty of Veterinary Medicine, “Ion Ionescu de la Brad” Iasi University of Life Sciences, 700489 Iasi, Romania; mspatarufmv@yahoo.com; 7 Department of Biostructure and Animal Physiology, Faculty of Veterinary Medicine, Wrocław University of Environmental and Life Sciences, 51-631 Wrocław, Poland; karolina.gozdziewska-harlajczuk@upwr.edu.pl

**Keywords:** calcaneus morphology, geometric morphometrics, hair goat, tarsal bones, locomotion, comparative anatomy, veterinary anatomy

## Abstract

The calcaneus plays a critical role in balance, locomotion, and muscle attachment, making it a key structure for biomechanical adaptations. This study examined interspecies differences between sheep and goats, as well as intraspecies variations among different sheep breeds, in calcaneus morphology. A total of 128 right calcanei were analyzed using 3D geometric morphometric methods to allow detailed assessments of calcaneus shape and size. Among sheep, the Hamdani sheep displayed the largest calcaneus among the breeds, distinguishing them significantly from the others. In contrast, goats had a bilaterally narrower calcaneal body, while sheep featured a more pronounced distal portion that articulates with the talus and a deeper articular surface. Additionally, the sustentaculum tali were more developed in sheep. The bilaterally compressed calcaneal body in goats likely reflects their agility and ability to navigate steep, rocky terrains. In contrast, sheep’s broader and more robust calcaneus may support their grazing lifestyle on flatter terrains, emphasizing their stability and weight-bearing capacity. These findings highlight the functional significance of calcaneal morphology in the locomotor strategies and physical capabilities of goats and sheep, providing valuable insights for comparative anatomy and veterinary science.

## Simple Summary

Sheep and goats are closely related species, but their skeletal structures may show distinct differences. This study focuses on the anatomical variations of the calcaneus in sheep and goats. Using geometric morphometric methods, the shape and size of the calcaneus in different sheep breeds and goats were analyzed. The results revealed that goats have a narrower and more compressed calcaneus, whereas sheep possess a broader and more developed structure. These differences contribute to species identification and classification in anatomical and taxonomic studies. The results provide reference data for veterinary anatomy and taxonomic studies, helping to distinguish species based on skeletal features.

## 1. Introduction

Bovids are a diverse group of ruminant mammals characterized by their hooves and unbranched, hollow horns [1]. The family *Bovidae* (*Artiodactyla* and *Ruminantia*) consists of approximately 140 species classified into 45 genera [2]. Its members are widely distributed across much of the world compared to other mammalian families. Sheep (*Ovis aries*) and goats (*Capra hircus*) belong to the tribe *Caprini* within the subfamily *Caprinae*, a subgroup of the *Bovidae* [3,4]. Due to their close taxonomic relationship, the skeleton of these two species exhibits significant similarities [5]. Investigating the similarities and differences in the functional morphology of the bones and overall body structure in closely related mammalian species can provide important information about the adaptive changes these species have undergone. Distinguishing between the skeletal remains of these species is particularly important in various scientific disciplines, including comparative anatomy, taxonomy, biomechanics, and zooarchaeology.

There are notable differences in the biomechanics of locomotion between sheep and goats. Goats are more adept at climbing compared to sheep [6]. Their lighter weight and narrow body profile facilitate their ability to navigate and climb narrow ledges [7]. Additionally, they have relatively larger forequarters, including proportionally long necks and well-developed shoulder muscles [8]. In contrast, sheep are generally more inclined to jump than goats [9]. The generation of a propulsive force plays a critical role in the success of the push-off phase during climbing, with the hind limbs, particularly the tarsal joint, being of key importance in providing this advantage [9]. The tarsal region is integral to this movement, with specific bones contributing to effective locomotion. The talus, located in the proximal row of the tarsus, connects the foot to the tibia and is a robust bone adapted to bear and transmit body weight [10]. The calcaneus, the largest tarsal bone, is located caudolateral to the talus (astragalus) and plays a crucial role in balance and locomotion by supporting body weight, articulating with the talus, and serving as an attachment site for various muscle tendons [11,12,13]. It has articular surfaces for articulation with both the talus and os tarsale IV. In ruminants, it also features a third surface that articulates with the os malleolare. Its free proximal end, known as the tuber calcanei, serves as the attachment site for the tendo calcaneus communis. Given these biomechanical differences, it is plausible that morphological variations in the calcaneus exist between species.

Geometric morphometrics is a powerful approach for studying morphological diversity [14,15]. It employs statistical tools to analyze shape differences by focusing on the positions of anatomical landmarks on biological structures. These landmarks are evaluated through principal component analysis, identifying the most significant vectorial changes contributing to shape variation [16,17]. Unlike linear morphometric methods, geometric morphometrics can isolate the shape from the size, position, and orientation, enabling a more precise understanding of morphology [18]. With advancements in technology, automated landmarking techniques have become increasingly popular [19,20,21]. These methods streamline the process by objectively placing landmarks across complex structures, saving time and reducing potential human error. Geometric morphometrics can be applied in both two-dimensional and three-dimensional contexts. The 3D approach, in particular, provides detailed insights into both visual and quantitative aspects of structural shape. This makes it one of the most effective methods for investigating subtle morphological differences, even in highly intricate structures like the calcaneus.

This study aims to compare the calcaneus morphology between sheep breeds and one goat breed, investigating intraspecies variations among sheep breeds and interspecies differences between sheep and goats. Various studies utilizing the calcaneus in sheep and goats are available in the literature [22,23,24,25,26,27]. Osteometric methods, particularly in zooarchaeological research, have frequently employed the calcaneus for species identification [22,23,24,25]. These studies have also been used in taxonomic research to obtain results related to species-specific size differences. Research focusing on the structure of the shape has primarily examined the two-dimensional morphology of the calcaneal bone, revealing interspecies differences [26,27]. In contrast to previous studies, this research aims to compare the three-dimensional morphology of the calcaneus in sheep and goats, providing a more detailed comparison of the calcaneal shape interspecies. Specifically, this study aims to utilize automated landmarking techniques to objectively and comprehensively map the entire structure of the bone, enabling the identification and analysis of subtle morphological differences that may not be captured through traditional methods or two-dimensional geometric morphometric analyses. By adopting this approach, we aim to enhance the understanding of species-specific adaptations in the calcaneus and contribute to the broader fields of comparative anatomy and functional morphology.

## 2. Materials and Methods

### 2.1. Samples

This study utilized calcaneus samples collected from Türkiye: 128 right calcanei from 1-year-old male individuals were analyzed to ensure homogeneity in age and sex. The samples were obtained from Akkaraman sheep (*n* = 37), Morkaraman sheep (*n* = 18), Hamdani Sheep (*n* = 20), and Hair goats (*n* = 53) from a slaughterhouse. After collection, all specimens were transported to the Department of Anatomy at the Faculty of Veterinary Medicine, Istanbul University-Cerrahpaşa, for preparation and analysis. Bones were carefully extracted from the tarsal region and subjected to a series of preparation steps to ensure the cleanliness and integrity of the samples. First, the bones were boiled to remove any remaining soft tissues. Subsequently, they were immersed in hydrogen peroxide to eliminate residual adipose tissue and ensure optimal surface clarity for scanning. Finally, the samples were air-dried and stored under controlled conditions until further processing.

The calcanei were digitalized using a Shining EinScan-SP 3D scanner (Shining 3D, Hangzhou, China) equipped with a camera resolution of 1.3 megapixels (DC 12-volt, 3.33 ampere). The scanning process utilized a turntable to ensure complete and consistent capture of the bone surfaces from all angles. A resolution of 1280 × 720 was applied to achieve high-quality images, and the data were processed using EXScan software (version 3.1.2.0, Shining 3D, Hangzhou, China). This software facilitated the generation of detailed 3D models by integrating individual scans into a cohesive whole. The finalized 3D models of the calcanei were saved in PLY file format, which preserves high-resolution geometric and texture data, making the models suitable for subsequent geometric morphometric analyses.

**Figure 1:**
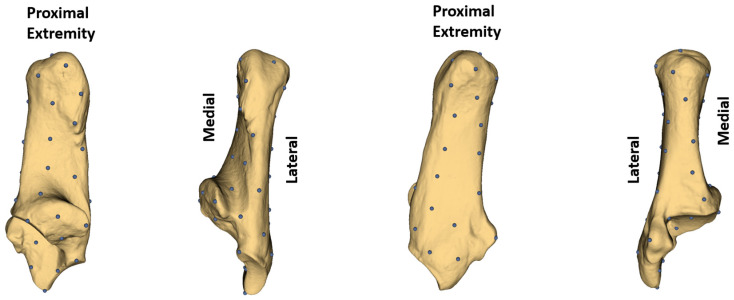
Landmarks.

To comprehensively represent the complex morphology of the calcaneus, automated landmarking was performed using the Slicermorph add-on in 3D Slicer (version 5.5.0) [28]. The ‘PseudoLMGenerator’ module was employed to create a set of 47 equidistant pseudo-landmarks (Figure 1) across the entire surface of the calcaneus [29]. These landmarks were transferred to all samples using the Automated Landmarking through Point Cloud Alignment and Correspondence Analysis (ALPACA) module, which applied point cloud alignment and shape-deformable mesh registration [30]. This method ensured accurate and consistent landmark placement across all specimens, resulting in 47 landmarks per calcaneus dataset for further morphometric analysis.

### 2.2. Geometric Morphometrics

The 3D landmark coordinates obtained from the calcanei were analyzed using RStudio (version 2024.12.0+467, 9 January 2024) software [31]. The analysis focused on identifying and quantifying shape variations across the three groups, with an emphasis on interspecies and intraspecies comparisons. The ‘Geomorph’ package (v.4.0.4) was employed for all geometric morphometric analyses [31,32]. To standardize the specimens and isolate isometry-free shape variation, Generalized Procrustes Analysis (GPA) was applied. This process aligned, scaled, and rotated all specimens to a standard coordinate system, ensuring that the observed differences represented actual shape variation rather than size or orientation effects.

Centroid size (CS) was calculated to quantify the overall size of each specimen based on its landmark configuration. First, the centroid (geometric center) of each specimen was determined by averaging the coordinates of all landmarks. Then, the CS value was obtained by summing the squared distances of each landmark from this centroid and taking the square root of this sum.

Pairwise comparisons of the squared means of centroid sizes were performed to assess size differences among the groups. Procrustes ANOVA was conducted to evaluate the influence of size on shape (allometry) and to determine whether the relationship between size and shape differed significantly among the groups. Additionally, group-specific allometric slopes were analyzed separately to examine how size-related shape changes varied between sheep and goats.

Principal component analysis (PCA) was employed to explore patterns of morphological variation. The PCA extracted major axes of shape variation, with the first two components (PC1 and PC2) representing the most significant aspects of shape differences across the dataset. These components were visualized as morphometric grids and 3D reconstructions to facilitate interpretation. Shape changes associated with PC1 and PC2 were analyzed using SlicerMorph (3D Slicer software, version 5.5.0), providing explicit visual representations of morphological variation [28].

ALPACA can also perform alignment between two sample meshes, enabling effective visualization of differences between 3D models [30]. This study visualized the differences between sheep and goat calcaneus models using representative sample models. First, separate GPA analyses were conducted for sheep and goats, and the individuals closest to the average shape for each species were identified by analyzing Procrustes distances. These two representative models (one sheep and one goat) were then aligned using point cloud alignment, and the meshes were overlaid to illustrate their morphological differences effectively.

To statistically test for shape differentiation between groups, Procrustes ANOVA was repeated using the shape data derived from GPA. The significance of group differences was assessed using permutation tests with 10,000 iterations to ensure robust results. Finally, pairwise comparisons were conducted to determine the specific morphological traits distinguishing each group.

All anatomical terms used in this study follow the Nomina Anatomica Veterinaria (NAV, 2017) to ensure standardization and consistency in veterinary anatomical nomenclature [33].

## 3. Results

### 3.1. Shape

The ANOVA results were used to evaluate the shape differences between the goat and sheep groups and pairwise comparisons among sheep subgroups (Hamdani, Akkaraman, Morkaraman). The proportion of variance explained (R^2^) was determined using the analysis of variance (ANOVA) method. The study between the goat and sheep groups revealed statistically significant shape differences (F = 26.257, *p* = 0.001). It was found that group differences explained 17.2% of the total variance (R^2^ = 0.172). This result indicates distinct differences in the calcaneus bone morphology between goats and sheep (Table 1).

Pairwise comparisons among sheep subgroups also revealed significant differences. In the comparison between Akkaraman and Hamdani groups, 4.0% of the shape variance (R^2^ = 0.040) was explained by group membership, and the difference was statistically significant (F = 2.2928, *p* = 0.004). For the Akkaraman and Morkaraman groups, group differences accounted for a higher proportion of variance (5.5%, R^2^ = 0.055), and the difference was significant (F = 3.1135, *p* = 0.001). Similarly, the comparison between the Hamdani and Morkaraman groups showed that 5.5% of the variance (R^2^ = 0.055) was attributable to group membership, with a statistically significant difference (F = 2.08, *p* = 0.006).

In conclusion, the shape differences between goats and sheep were evident, while the differences among sheep breeds were significant but relatively moderate.

### 3.2. Size and Allometry

Goats had a mean centroid size of 121.53, reflecting a generally smaller size than sheep, which exhibited a mean centroid size of 126.57. Among the sheep subgroups, Hamdani sheep had the largest mean centroid size at 133.00, followed by Morkaraman sheep at 124.25 and Akkaraman sheep at 124.23. The analysis of the centroid size revealed significant differences between goats and sheep and among the sheep subgroups (Table 2). The comparison between goats and sheep showed a statistically significant difference in centroid size (F = 13.33, *p* = 0.002, R^2^ = 0.096), indicating that the difference between these two groups explains 9.6% of the variation in centroid size. This result suggests an evident distinction in overall size between the two primary groups.

Within the sheep subgroups, significant variations in centroid size were observed. The comparison between Akkaraman and Hamdani sheep revealed a statistically significant difference (F = 17.66, *p* = 0.001, R^2^ = 0.243), with 24.3% of the variation in centroid size attributed to differences between these breeds. In contrast, no significant difference was observed between Akkaraman and Morkaraman sheep (F = 0.0003, *p* = 0.987, R^2^ ≈ 0), indicating that these two subgroups are similar in size. The analysis of Hamdani and Morkaraman sheep also showed a significant difference (F = 9.99, *p* = 0.002, R^2^ = 0.217), with 21.7% of the variation in centroid size explained by the difference between these breeds.

The allometric regression analysis revealed significant relationships between the centroid size and shape in goats and sheep. For goats, the study showed that 4.65% of the shape variation (R^2^ = 0.0465) was explained by size, with a significant *p*-value of 0.004, an F-statistic of 2.487, and a Z-score of 2.4916. Similarly, for sheep, 3.29% of the shape variation (R^2^ = 0.03288) was attributed to size, with a *p*-value of 0.001, an F-statistic of 2.4818, and a Z-score of 3.3148. These results indicate that size has a statistically significant but modest influence on the shape variation in both groups. However, in goats, the relationship between the size and shape appears slightly stronger, as larger individuals tend to exhibit a relatively more elongated and slenderer calcaneus. This suggests that allometric effects are more pronounced in goats compared to sheep.

### 3.3. Shape Variation

**Figure 2:**
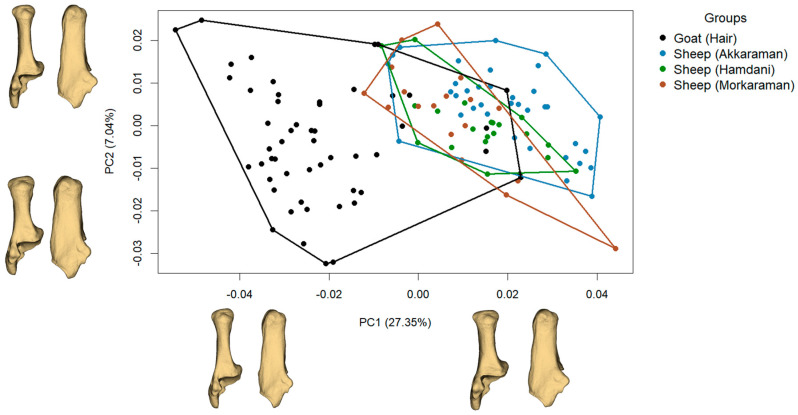
Distribution of PC1 and PC2 in the morphospace for the calcaneus, along with 3D visualizations of positive and negative shape changes associated with each principal component.

The principal component analysis (PCA) results reveal evident distinctions between groups and subgroups based on PC1 and PC2 values (Figure 2). The average PC1 value for the Goat group is −0.0214, whereas, for the Sheep group, it is 0.0151. Similarly, the average PC2 values are −0.0038 for Goat and 0.0027 for Sheep. Subgroup-wise, Akkaraman Sheep exhibit the highest average PC1 value (0.0195), followed by Hamdani Sheep (0.0139), Morkaraman Sheep (0.0075), and Hair Goat (−0.0214). For PC2, Akkaraman Sheep again show the highest average value (0.0042), with Morkaraman Sheep (0.0026), Hamdani Sheep (0.0001), and Hair Goat (−0.0038) following. A statistical analysis confirms significant PC1 and PC2 value differences between the Goat and Sheep groups. The ANOVA for PC1 yielded a highly significant F value of 183.2 (*p* < 0.001), indicating a robust distinction in PC1 values between these groups. PC2 primarily reflects individual variation rather than consistent group-level differences. The variation for PC2 appears more attributable to individual morphological traits rather than shared group characteristics.

A negative PC1 value represented a narrower calcaneal body in shape. As the PC1 value increased, the calcaneus exhibited a broader body. The calcaneal tuberosity, where the Achilles tendon attaches, was more developed with positive PC1 values. The sustentaculum tali also appeared more developed along the positive PC1 axis. For negative PC2 values, the primary shape change was observed in the calcaneal tuberosity. A narrow (bilaterally compressed) body and a more pronounced calcaneal tuberosity were characteristic of negative PC2. In contrast, with positive PC2 values, the calcaneal tuberosity and the calcaneal body displayed a similar width level.

**Figure 3:**
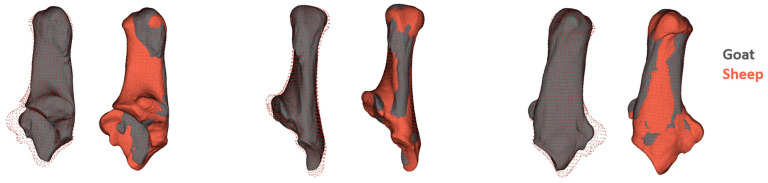
Visualization of morphological differences between goat and sheep calcanei using overlaid point cloud and rigidly registered models. Sheep specimens exhibit a broader calcaneal structure compared to goat specimens. After aligning and overlaying the goat and sheep models with size differences removed, the broader sheep model obscures certain features of the goat calcaneus. Therefore, in the figure, the sheep specimens are visualized as a “point cloud” and as “rigidly registered” models to provide a comprehensive depiction.

The visual results generated from the PC1 findings and the models of the two average individuals were consistent and complementary (Figure 3). The body of the calcaneus (*corpus calcanei*) appeared similar in shape when viewed from the lateral and medial aspects in both goats and sheep. However, in goats, this section was bilaterally compressed. The distal portion of the calcaneus, which articulates with the talus, was more pronounced in sheep. Additionally, the articular surface of the talus that interfaces with the calcaneus was deeper in sheep. The sustentaculum tali, supporting the talus, exhibited a more developed morphology in sheep. In contrast, the sulcus for the passage of the flexor digitorum profundus muscle was deeper in goats.

## 4. Discussion

The results of this study reveal significant morphological differences between goat and sheep calcanei. The ANOVA results demonstrated clear shape and size differences between the two groups, with sheep calcanei generally exhibiting larger centroid sizes compared to goats. Among sheep subgroups, Hamdani sheep displayed the largest centroid size, followed by Akkaraman and Morkaraman breeds, indicating potential breed-specific size adaptations. These size differences likely reflect varying environmental and functional demands among the breeds, with Hamdani sheep, known for their larger body mass, exhibiting more robust calcanei. The PCA analysis further reflected these differences, where goats displayed narrower calcaneal bodies and bilaterally compressed shapes. At the same time, sheep exhibited broader distal structures, particularly in the sustentaculum tali and calcaneal tuberosity.

The morphological differences observed in the calcaneus between goats and sheep suggest adaptations to their locomotor and ecological demands. The bilaterally compressed corpus calcanei in goats and a narrower talus bone [34] reflect their agility and ability to navigate steep, rocky terrains, where a streamlined skeletal structure may facilitate better mechanical leverage and stability. Biomechanical studies have shown that narrower phalanges or hooves, compared to more rounded structures, exhibit better resistance to slipping, providing a significant advantage during climbing [35,36]. Although these studies focused on hooves or phalanges, it is reasonable to hypothesize that the calcaneus and talus, integral to the same functional unit, may exhibit structural adaptations similar to these lower skeletal elements, suggesting that goats’ narrower calcaneus and talus may be an adaptation to enhance stability and grip on rugged surfaces. In contrast, the broader and more developed corpus calcanei in sheep and a comparatively wider talus may be linked to their grazing lifestyle on relatively flatter terrains, requiring less maneuverability but more significant support for prolonged weight-bearing. These findings align with Köhler’s [37] classification of ruminant locomotor adaptations, which differentiates between species adapted to open environments and those specialized for more rugged terrains. In open-habitat species, the calcaneus tends to be more elongated, with a larger and more convex talar articulation, promoting efficient forward propulsion. Meanwhile, in species adapted to rough terrains, the calcaneus is more robust, with a less pronounced articular surface, enhancing stability and lateral flexibility for movement across uneven landscapes. Although our study focused on more closely related subgroups compared to Köhler’s broader classification, the results remain consistent and mutually supportive, reinforcing the relationship between the calcaneus morphology and habitat-driven locomotor specialization in ruminants.

The morphological differences in the calcaneus between ruminants adapted to open and forested environments align with both Köhler [37] and Barr [38]. Köhler’s ecological classification emphasized skeletal adaptations related to habitat, while Barr’s [38] quantitative analyses demonstrated that calcaneus length and shape exhibit strong allometric scaling and habitat-driven modifications. Barr’s findings show that open-habitat bovids, which rely on cursorial locomotion, tend to have relatively shorter calcanei to optimize plantarflexion speed, whereas forest-adapted bovids have longer calcanei, enhancing their ability to navigate complex terrains. Additionally, Barr [38] highlighted those elongated calcanei are particularly associated with stotting behavior, an energy-demanding leaping motion seen in Antilopini as a predator avoidance strategy. Similarly, goats, which frequently engage in jumping and climbing behaviors, exhibit a more elongated and slenderer calcaneus compared to sheep, likely enhancing their propulsion efficiency and maneuverability on rugged terrains. These observations further reinforce the notion that the primary function of the calcaneus is not solely weight-bearing but rather providing an effective lever for propulsion. This biomechanical role is crucial for species requiring rapid acceleration or enhanced stability in complex environments.

The more pronounced sustentaculum tali in sheep likely reflect their greater body weight than goats. This structure is critical in stabilizing the talus and supporting the forces transmitted through the hindlimb during locomotion. In sheep, the enhanced development of the sustentaculum tali could accommodate the increased mechanical demands associated with bearing more weight while walking or standing for extended periods during grazing. In contrast, goats, which are generally lighter and more agile, exhibit a relatively less developed sustentaculum tali but compensate with adaptations such as a deeper sulcus for the flexor digitorum profundus muscle. This feature facilitates better tendon protection and greater flexor efficiency, which is crucial for climbing and navigating steep, uneven terrains.

The observed differences in the calcaneus also raise intriguing questions about the developmental pathways that have shaped these structures in goats and sheep. The shared basic morphology of the calcaneus reflects their common evolutionary ancestry as members of the Bovidae family [38]. However, the variations in specific regions, such as the corpus calcanei, distal articular surface, and sustentaculum tali, indicate selective pressures that have acted on these species to optimize their skeletal system for distinct environmental challenges. Further research integrating biomechanical analysis and genetic studies could provide deeper insights into the evolutionary drivers of these morphological differences.

Building upon Lloveras’s findings [27], which examined calcaneal morphology using two-dimensional analysis, our study provides a more detailed perspective by applying three-dimensional geometric morphometric techniques. Both studies consistently highlight the significant morphological differences in the calcaneus of sheep and goats, particularly in regions such as the calcaneal tuber, sustentaculum tali, and articular surfaces. Consistent with Lloveras’s observations, our study found that sheep calcanei feature a more developed calcaneal tuber and sustentaculum tali, adaptations likely suited to their grazing lifestyle on flat terrains requiring stability and weight-bearing. Conversely, goats exhibit a more compact and streamlined calcaneal structure, with the malleolus articular surface being shorter and projecting anteriorly. However, one of the most notable findings of our study was the bilaterally compressed body of the goat calcaneus. Lloveras’s two-dimensional analysis did not capture this feature, which relied on medial-view images. In our analysis, the PC1 variation emphasized this important morphological characteristic of goats, further underscoring the value of three-dimensional methods. The three-dimensional analysis in this study allowed us to detect and quantify subtle geometric variations that are challenging to observe in two-dimensional projections. This highlights the critical advantage of three-dimensional techniques in providing a holistic understanding of morphological adaptations, enabling a more accurate interpretation of functional and ecological differences between species.

## 5. Conclusions

This study identified species-specific anatomical variations in the calcaneus of goats and sheep using geometric morphometric methods. Shape variation was found to be effective in distinguishing between these two species. Geometric morphometric techniques enabled a precise and detailed understanding of these variations, allowing for the detection of subtle shape differences that traditional morphometric methods might overlook. This method, which has been increasingly used in veterinary anatomy and taxonomy studies in recent years, provides a more comprehensive evaluation of skeletal morphology, making significant contributions to anatomical and taxonomic research [39,40,41].

## Figures and Tables

**Table 1:** Results of Procrustes ANOVA for shape variation among goat and sheep groups.

Comparison	Sum of Squares	Mean Square	F Value	*p* Value
Goat vs. Sheep	0.044090	0.044090	26.2570	0.001
Akkaraman vs. Hamdani	0.003614	0.003613	2.2928	0.004
Akkaraman vs. Morkaraman	0.004827	0.004827	3.1135	0.001
Hamdani vs. Morkaraman	0.003426	0.003426	2.0800	0.006

**Table 2:** Results of ANOVA for centroid size variation among goat and sheep groups.

Comparison	Sum of Squares	Mean Square	F Value	*p* Value
Goat vs. Sheep	789.3	789.27	13.33	0.002
Akkaraman vs. Hamdani	999.7	999.66	17.66	0.001
Akkaraman vs. Morkaraman	0.01	0.0066	0.0003	0.987
Hamdani vs. Morkaraman	725.6	725.60	9.99	0.002

## Data Availability

The original contributions presented in this study are included in the article. Further inquiries can be directed to the corresponding author (O.G.).
